# ^18^F-FDG PET/CT versus bone marrow biopsy in detecting bone marrow infiltration in initial staging of pediatric lymphoma

**DOI:** 10.1186/s41824-024-00200-0

**Published:** 2024-04-15

**Authors:** Nahla Bashank, Seham Sharef, Taha Zaki Mohran, Maha Khalil

**Affiliations:** 1 Department of Clinical Oncology and Nuclear Medicine, Assiut University Hospital, Assiut University, Assiut, Egypt; 2 Radiotherapy and Nuclear Medicine Unit, South Egypt Cancer Institute, Assiut University, Assiut, Egypt

**Keywords:** ^18^F-FDG, PET/CT, Bone marrow biopsy, Bone marrow infiltration, Pediatric lymphoma, Initial staging

## Abstract

**Background:**

To evaluate the efficacy of PET/CT using^18^F-FDG (^18^F-fluorodeoxyglucose) as a radiotracer compared to conventional bone marrow biopsy (BMB) in detecting infiltration to bone marrow (BM) in pediatric patients with lymphoma at the time of initial diagnosis.

**Methods:**

66 pediatric patients with lymphoma (47Hodgkin’s and 19non-Hodgkin’s lymphoma) were referred for initial staging by^18^F-FDG PET/CT study. All patients underwent bilateral iliac BMB and ^18^F-FDG PET/CT scan with no more than 2 weeks interval in-between. Follow-up for at least 6 months was used as a reference standard to compare diagnostic performance between two modalities in detecting bone marrow infiltration (BMI).

**Results:**

Sensitivity, specificity, accuracy, as well as positive and negative predictive values of ^18^F-FDG PET/CT in detecting BMI were (80%, 86%, 85%, 63%, and 94%) in contrast to BMB (80%, 53%, 59%, 33%, and 90%) respectively. ^18^F-FDG PET/CT was concordant to BMB in 39/66 patients (59%).

**Conclusion:**

^18^F-FDG PET/CT was more accurate and specific, with higher predictive values than BMB in detecting BMI during initial staging of pediatric lymphoma. In most pediatric lymphoma patients, ^18^F-FDG PET/CT can be used instead of BMB to determine BMI during their initial staging process.

## Background

Malignant lymphoma is recognized as one of the most prevalent forms of hematological malignancy worldwide (Jhanwar and Straus 2006). Lymphomas can be classified into two main categories: Hodgkin’s lymphoma (HL) and non-Hodgkin’s lymphoma (NHL). BMI is detected in around 15% of recently diagnosed cases of diffuse large B-cell subtype of NHL, with a lower occurrence rate observed in HL(Howell et al. 2002). Proper staging is crucial to optimize therapy and minimize the occurrence of severe adverse effects and toxicity (Connors 2005).

The conventional diagnostic approach for BMI has been to perform a marrow biopsy -from either of the iliac crests (Howell et al. 2002, Brusamolino et al. 2009). Nevertheless, BMB has limitations due to its aggressive nature and its ability to provide information on bone marrow involvement from only one specific location. This limitation is not informative when the BMI is scattered or patchy (Haddy et al. 1989).

Currently, ^18^F-fluorodeoxyglucose positron emission tomography/computed tomography (^18^F-FDG PET/CT) is successfully employed for staging and monitoring of patients with NHL and HL (Almuhaideb et al. 2011; Radford et al. 2015). Multiple investigations have verified that ^18^F-FDG PET/CT effectively detects BMI and produces similar results to BMB(Pelosi et al. 2008). Therefore, it is assumed to have a complementary function in diagnosing BMI(Hong et al. 2012).

Therefore, the current study aimed to compare the overall diagnostic performance of ^18^F-FDG PET/CT and its concordance with conventional BMB in detecting BMI in the initial assessment of pediatric patients with lymphoma.

## Patient and methods

All pediatric patients under the age of 18 years with pathologically confirmed Hodgkin’s or non-Hodgkin’s lymphoma (prior treatment) who underwent ^18^F-FDG PET/CT study and BMB for initial staging with no more than 2 weeks interval between both modalities. Patients with more than 2 weeks interval or started chemo or radiotherapy before BMB or ^18^F-FDG PET/CT study were eliminated.

This study was approved by the Institutional Review Board at the Faculty of Medicine, Assiut University (IRB No., 04-2022-200018) with a clinical trial number (NCT0556898).

## Bone marrow biopsy

### Preparation

The patient was put prone on the operation table with the posterior superior iliac crest exposed. The skin was then cleansed. The location of the biopsy was determined by palpating the iliac crest. An antiseptic solution was used to sterilize the puncture site. The area was anesthetized with local anesthetic. Then, trephine BMB was taken from the posterior iliac crest bilaterally.

### BMB interpretation

After decalcification, the specimen was stained with hematoxyline and eosin and then examined for marrow infiltration by two pathologists unaware of ^18^F-FDG PET/CT results.

## ^**18**^**F-FDG PET/CT study**

### Preparation

Fasting for at least 4 h is required before the study. Following an FDG injection of 0.14-0.3mCi/kg (5-11MBq/Kg), the patient was placed in a warm, dark and quiet area.

Serum glucose level below 150 mg/dl was mandatory at time of radiotracer injection.

### Technique

PET images were acquired from the skull vertex to the middle of the thigh 60–90 min later. Simultaneous non-contrast CT scan was performed for attenuation correction and fusion with PET images to permit anatomical localization. The images were acquired on a hybrid PET/CT scanner (Biograph m CT flow; Siemens Healthineers) with 16 slice CT component. PET images were employed immediately after CT images acquisitions (5–7 bed positions) with an acquisition time of 1 to 2 min for each bed position, then reconstructed in transverse, sagittal, and coronal views using an iterative algorithm.

### Interpretation

The study was examined and interpreted by experienced physicians on the manufacturer’s workstation (Synovia Siemens Healthcare).

### Qualitative assessment

Any focal/multifocal lesions or diffuse heterogeneous FDG uptake in BM greater than liver reference uptake was considered abnormal. Revision of CT images for corresponding alterations was done. Diffuse homogenous BM uptake equal to or less than liver uptake was considered reactive hyperplasia and negative for BMI.

### Quantitative assessment

A region of interest (ROI) was created over the iliac bone posteriorly to obtain SUV _max_ for BM uptake (BM SUV_max_). A second ROI was created over the liver (liver SUV_max_) as a benchmark for uptake.

The ratio between BM SUV_max_ and liver SUV_max_ was calculated and recorded.

## Data analysis and interpretation

### The reference standard

To validate the results, follow-up for at least 6 months was used as a gold standard through other radiological, clinical and laboratory data in addition to follow-up ^18^F-FDG PET/CT to confirm or exclude BMI.

**The study was considered Positive for BMI** when there is abnormal BM uptake, whether focal or diffuse heterogeneous marrow uptake, greater than the liver uptake. The study was considered Negative for BMI when diffuse homogenous BM uptake is equal to or less than the liver uptake.

### True positive results

If both^18^F-FDG PET/CT and BMB were positive and follow-up of the patient, whether clinical or radiological, was positive for BMI.

### True negative results

If both^18^F-FDG PET/CT and BMB were negative and follow-up was negative for BMI.

### False-positive results

Patients with positive or equivocal results in BMB or ^18^F-FDG PET/CT study yet negative for BMI in follow-up.

### False-negative results

Patients with negative results in BMB or ^18^F-FDG PET/CT study, yet BMI proved in follow-up by further evaluation.

**Equivocal Results** were considered when there was unexplained diffuse homogenous marrow uptake greater than liver uptake in ^18^F-FDG PET/CT or inconclusive results in BMB that necessitated further investigation by immunophenotyping.

Equivocal results were considered positive in statistical analysis.

**Concordant Results** considered if both modalities had similar results, whether positive or negative.

**Discordant Results** were considered when ^18^F-FDG PET/CT was positive for BMI while BMB was negative and vice versa.

### Statistical analysis

IBM SPSS version 26 (SPSS Inc., Chicago, IL) was used for data analysis. Qualitative variables were represented as frequencies and percentages, while quantitative data was expressed as means ± standard deviations (SD). SUV_max_ over the iliac bone was used to measure the uptake of BM (SUV_BM_). A ratio between iliac SUV_max_ and liver SUV_max_ was obtained to compare the BM uptake between positive and negative cases of BMI. The diagnostic performance indices for ^18^F-FDG PET/CT and BMB were generated with 95% confidence intervals for the whole study and separately for HL and NHL.

Agreement between BMB and ^18^F-FDG PET/CT results was calculated using Cohen’s k computation for the entire study and separately for HL and NHL.

## Results

This study was conducted on 66 pediatric patients (54 male and 12 female) with recently diagnosed lymphoma (47 HL and 19 NHL). The mean age was 10 ± 3.8, median 14 (range 4–18 years). B symptoms (fever > 38 C, night sweating, and weight loss) were noted in 22 patients (33.3%); 20 cases of them had HL. Patients’ characteristics with different types of HL and NHL are shown in Table 1.

### The pattern of BM uptake

Most patients, 57 out of 66 cases (86.4%), had diffuse homogenous BM uptake, while the remaining 9 patients (13.6%) had focal or heterogeneous FDG uptake, as described in Table 2.

Only 8 out of 57 patients (14%) with diffuse BM uptake were positive for BMI (5 cases with NHL and 3 with HL), while 49 of them (86%) were negative for BMI and proved to have reactive hyperplasia.

6 cases had focal uptake at multiple sites (dorsal, lumber vertebrae, pelvic bones and both femora), and only one case with a heterogeneous pattern proved positive.

The last 2 cases with unifocal BM uptake were considered negative after follow-up.

**Table 1:** Patients^’^ Characteristics

	HL(*n* = 47)	NHL(*n* = 19)
**Age** **Range**	10.57 ± 3.8 14(4-8)	9 ± 3.7 10(5-15)
**SEX**		
**Male** **Female**	38 (81%) 9 (19%)	16(84.2%) 3(15.8%)
**Pathological subtype**	Classic 7 (14.9%) Mixed cellularity 20 (42.6%) Nodular sclerosing 16 (34%) Lymphocytic predominant 3 (6.4%) Lymphocytic depletion 1 (2.1%)	T cell lymphoma 2(10.5%) B cell lymphoma 9(47.4%) Burkett’s lymphoma 8(42.1%)
**Initial staging**		
**I** **II** **III** **IV**	1 (2.1%) 16 (34%) 23 (48.9%) 7 (14.9%)	1 (5.3%) 4 (21.1%) 9 (47.4%) 5 (26.3%)
**B symptoms**		
**Present** **absent**	20 (42.6%) 27 (57.4%)	2(10.5%) 17(89.5%)

**Table 2:** Pattern of BM Uptake

Pattern of FDG Uptake	Frequency	Percentage %
homogenous diffuse	57	86.4
heterogenous	1	1.5
unifocal	2	3.0
multifocal	6	9.1
Total	66	100.0

### Evaluation of bone marrow infiltration

BMI was excluded in 51 patients (77.3%) and detected in 15 (22.7%) of cases: 7HL (10.6%), 8 NHL (12.1%).

Out of 15 BMI, 9 cases were detected by both BMB and ^18^F-FDG PET/CT.

3 cases were detected by ^18^F-FDGPET/CT **only** yet with negative BMB (the uptake in BM was focal, and the biopsy was taken from a non-active site).

3 cases were detected by **BMB only** with negative ^18^F-FDG PET/CT due to diffuse bone marrow uptake interpreted as reactive hyperplasia. Both modalities detect BMI in 12 out of 66 patients, with a detection rate of about 18%.

### Concordance and discordance between 18 F-FDG PET/CT and BMB

^18^F-FDG PET/CT and BMB were **concordant** in 39 patients (59%); 9 had positive results in both modalities, and 25 had negative results. While 5 patients had equivocal (false positive) results in both modalities proved to be negative after follow-up.

**Fig. 1:**
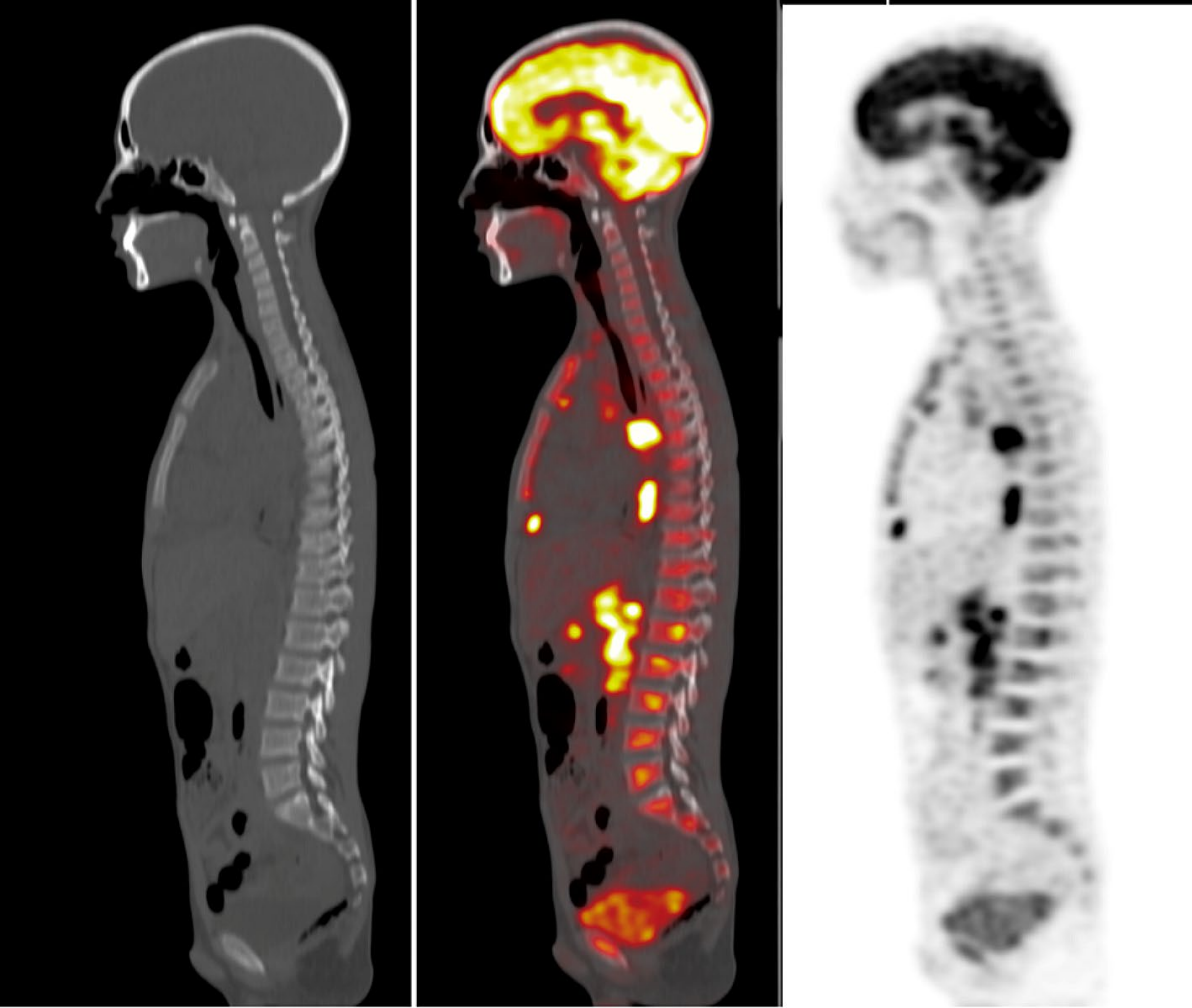
A 14-year-old male with Hodgkin’s lymphoma (nodular sclerosing subtype) PET, fused PET/ CT images and CT images in sagittal view showing focal BM uptake without corresponding CT changes. BMB was negative

**Discordant Results** were noticed in 27 patients (41%); 19 were true negative by ^18^F-FDG PET/CT with equivocal results in BMB. Another 3 cases with true positive findings in ^18^F-FDG PET/CT (1 case with focal BM uptake and 2 with diffuse heterogeneous uptake) yet negative BMB results (as biopsy not performed in the active sites) Fig. 1.

**Fig. 2:**
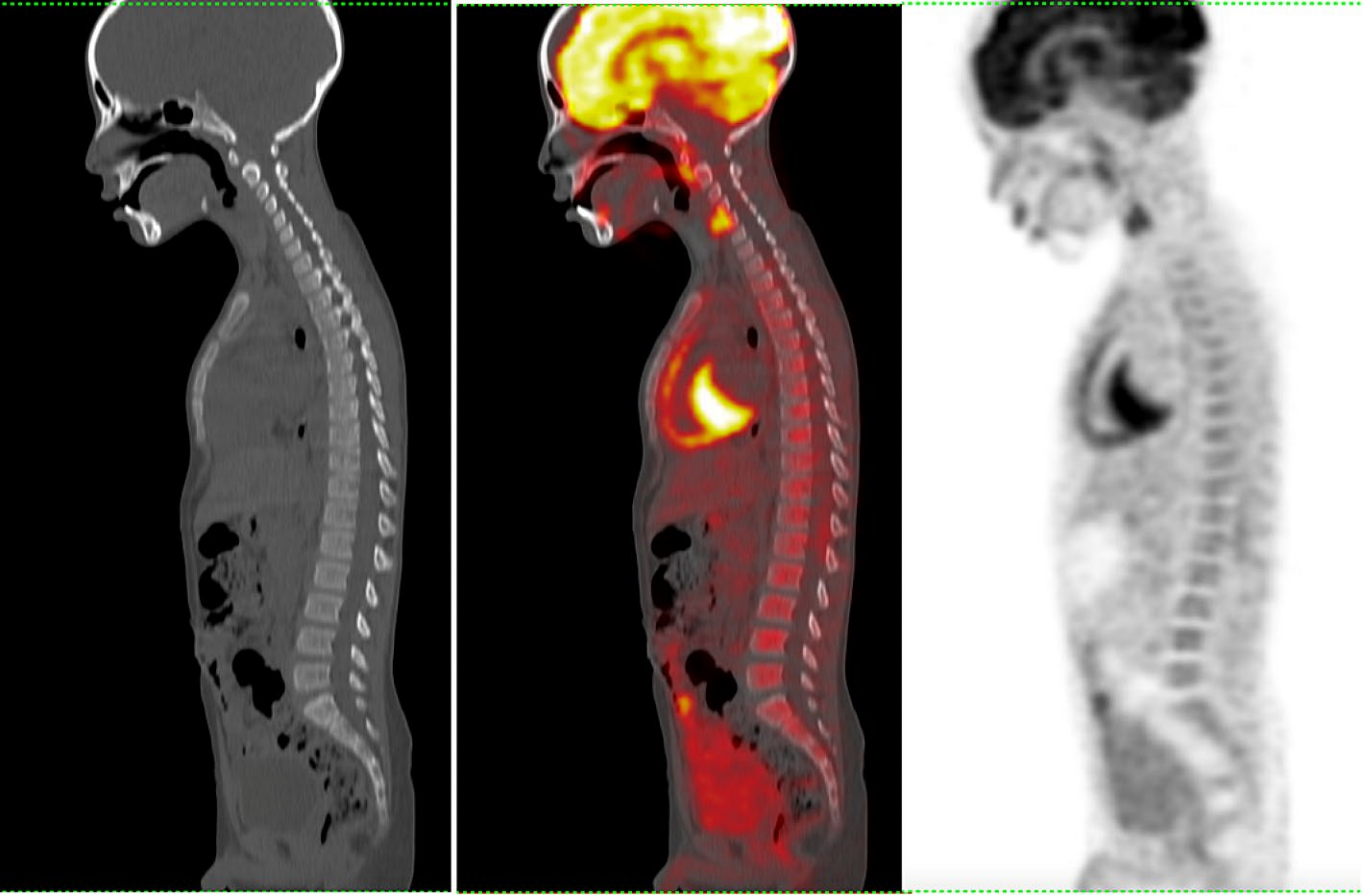
A 10-year-old male with Non-Hodgkin’s lymphoma (Burkett’s subtype) PET, fused PET/ CT images and CT images in sagittal view showing diffuse BM uptake. BMB was positive

3 cases also had false negative results in^18^F-FDG PET/CT yet positive for BMI by BMB because diffuse BM uptake equal to liver uptake was interpreted as reactive hyperplasia. Taking into consideration that diffuse uptake equal to or greater than liver uptake needs further evaluation by BMB, Fig. 2.

The last 2 cases had equivocal results (FP) in ^18^F-FDG PET/CT; however, BMI was excluded after BMB, as described in Table 3.

**Table 3:** Concordance/discordance between ^18^F-FDG PET/CT and BMB

		PET/CT results				Total
		TP	TN	FP	FN	
**BMB results**	TP	9	0	0	3	12
	TN	0	25	2	0	27
	FP	0	19	5	0	24
	FN	3	0	0	0	3
Total		12	44	7	3	66

### Equivocal results in BMB and^18^F-FDG PET/CT study in relation to the final diagnosis

9 cases with diffuse homogenous BM uptake greater than liver uptake were considered equivocal by ^18^F-FDG PET/CT; only 3 were negative for BMI after BMB, and 6 cases still had equivocal results.

30 cases were found to be equivocal by BMB, which necessitated further evaluation by ^18^F-FDG PET/CT. 20 cases were negative, and 4 were positive after ^18^F-FDG PET/CT study, while 6 were still equivocal, as shown in Table 4.

**Table 4:** Equivocal results in BMB and ^18^F-FDG PET/CT study

		^18^F-FDGPET/CT results			
		**Positive**	**Equivocal**	**Negative**	**Total**
**BMB Results**	**Positive**	4	0	2	6
	**Equivocal**	4	6	20	30
	**Negative**	2	3	25	30
**Total**		10	9	47	66

### Diagnostic performance of ^18^F-FDG PET/CT and BMB

^18^F-FDG PET/CT was more accurate (85% vs. 59%), more specific (86% vs. 53%) with higher negative and positive predictive value (94% vs. 90%), (63% vs.33%) than BMB, as illustrated in Table 5.

However, the sensitivity of both modalities was comparable to 80%. The increased sensitivity of BMB was due to bilateral iliac bone biopsy. ^18^F-FDG PET/CT was more sensitive, specific, and accurate in HL than NHL. The diagnostic performance of BMB is better in NHL than in HL.

**Table 5:** Diagnostic performance indices for ^18^F-FDG PET/CT and BMB in detection of BMI in Pediatric lymphoma

Pathology	Modality	Sensitivity	Specificity	PPV	NPV	Accuracy
**Hodgkin’s lymphoma**	PET/CT (95%CI)	86 (76–96)	90 (81–99)	60 (46–74)	97 (93–102)	89 (81–98)
	BMB (95%CI)	71 (59–84)	48 (33–62)	19 (8–30)	90 (82–99)	51 (37–65)
**Non-Hodgkin’s lymphoma**	PET/CT (95%CI)	75 (56–94)	73 (53–93)	67 (45–88)	80 (62–98)	74 (54–93)
	BMB (95%CI)	88 (73–102)	73 (53–93)	70 (49–91)	89 (75–103)	79 (61–97)
**Total**	PET/CT (95%CI)	80 (70–90)	86 (78–95)	63 (52–75)	94 (88–100)	85 (76–93)
	BMB (95%CI)	80 (70–90)	53 (41–65)	33 (22–45)	90 (83–97)	59 (47–71)

### Agreement between ^18^F-FDG PET/CT and BMB

Fair agreement (Kappa 0.33) was noticed between both modalities in patients with HL, while moderate agreement in NHL patients (kappa 0.45), Table 6.

### Semi-quantitative Assessment of BMI in positive and negative cases

The difference in the mean value of BM SUV_max_ between cases with positive BMI and negative cases was significant (1.4 vs.1.07), with a *P* value of 0.11.

The difference in the mean value of the ratio between BM SUVmax and liver SUV_max_ was also significant (1.3 vs. 0.86), with a *P* value of < 0.0001, as shown in Table 7.

**Table 6:** Agreement between ^18^F-FDG PET/CT and BMB.

Pathology						Value	Significance
**Hodgkin’s Lymphoma**		Measure of Agreement			Kappa	0.333	0.000
		N of Cases				47	
**Non-Hodgkin’s lymphoma**		Measure of Agreement			Kappa	0.455	0.002
		N of Cases				19	
**Total**		Measure of Agreement			Kappa	0.374	0.000
		N of Cases				66	

**Table 7:** Semi-quantitative Assessment between Positive and Negative Cases

		Mean	SD	*P* value
**BM SUV**_**max**_	**Positive**	1.4167	0.49047	0.11
	**Negative**	1.0726	0.43514	
**BM SUV**_**max**_ **ratio** **(BM SUV**_**max**_ **/liver SUV**_**max**_ **)**	**Positive**	1.33	0.42	0.000
	negative	0.86	0.32	

### Impact on patient staging

15/66 cases were proved to have BMI. Upstaging by^18^F-FDG PET/CT from stage II and III to stage IV was noticed in 2/66 cases (3%) by detection of BMI. Upstaging from stage III to stage IV by detection of BMI in BMB in another two cases (3%). 11 /66 cases (16.6%) had stage IV by nodal and/or extra-nodal affection, and the stage was confirmed by detection of BMI by both BMB and ^18^F-FDG PET/CT. In the remaining 51/66 cases, the stage was confirmed by ^18^F-FDG PET/CT by excluding BMI.

## Discussion

Evaluating BMI in lymphoma is crucial for determining the stage of the disease since its presence can advance the disease to stage IV. BMI is more frequent in pediatric NHL than in adults, 12.1% in our study (Chen et al. 2018).

BMB is an aggressive procedure that allows histopathologic analysis of a limited BM specimen obtained from posterior iliac crest. It can be painful and may result in problems like hemorrhage, infection, insufficient sample, and the need for re-biopsy. Blindly performing a unilateral iliac crest biopsy may result in a high rate of false negative bone marrow biopsies. On the other hand, ^18^F-FDG PET/CT is a noninvasive method that enables viewing of the entire BM in one setting (Elamir et al. 2020). This study aims to assess the role of ^18^F-FDG PET/CT in evaluating the infiltration to BM compared to BMB in 66 pediatric patients recently diagnosed with lymphoma.

The sensitivity of ^18^F-FDG PET/CT decreases when BMB is solely utilized as a reference standard (Vishnu et al. 2017). Therefore, we used follow-up findings, computed tomography (CT) changes, and/or magnetic resonance imaging (MRI) findings as a reference standard. This is a great advantage in our study characterizing it from other studies.

**Diffuse BM uptake** is widely debated in the majority of studies. According to most studies, diffuse uptake in the BM is linked to negative BM biopsies in cases of HL, however positive biopsies were commonly detected in cases of diffuse BM uptake in most cases of diffuse large B-cell lymphoma. This comes in agreement with our results in which the majority of patients with diffuse BM uptake, 49/57 (86%), had negative results with reactive BM hyperplasia; most of them had HL, while only 8/57 cases (14%) were positive for BMI; most of them had NHL, (Adams et al. 2014; El-Galaly et al. 2012).

A small proportion of cases in HL had a scattered pattern with BMI, as discovered by others (Salaun et al. 2009; Chiang et al. 2003).

In our study, 8 out of 15 positive cases of BMI had diffuse BM uptake. This is consistent with Cortés-Romera et al., who found that out of 9 cases with diffuse BM uptake, 4 had positive BMB findings (Cortés-Romera et al. 2014). Further study by Cerci et al. showed that 4 out of 18 NHL cases with diffuse BM uptake were positive in BMB. So, any case with diffuse BM uptake greater than or equal to liver uptake (equivocal findings) should be biopsied to establish etiology and differentiate between BMI and BM hyperplasia (Cerci et al. 2014). Also, Khan et al. reported that 5 patients with uniformly increased BM uptake were positive after biopsy (Khan et al. 2013).

We found that 6 out of 15 cases positive for BMI had focal uptake greater than liver uptake at various sites in dorsal, lumber vertebrae, pelvic bones and both femora and only 1 case with heterogenous diffuse pattern. The research conducted by Öner et al. found that widespread heterogeneous BM accumulations, which showed variations in composition, indicate a positive diagnosis for BMI (Öner et al. 2017).

Conversely, Lee et al. (Lee et al. 2012) discovered that a significant majority (90.9%) of patients with uniformly distributed increased BM activity exhibited positive BMB results in contrast to our study that showed only (14%) of patients with diffuse BM uptake were positive for BMI, while the majority had reactive BM hyperplasia.

^18^F-FDG PET/CT may not accurately detect minor illness with mild diffuse BM uptake, but BMB will yield positive results. In our study, diffuse BM uptake greater than liver uptake was considered equivocal results in ^18^F-FDG PET/CT (9 cases), necessitating further BMB to differentiate between BMI and BM reactive hyperplasia. Only 2 out of 9 equivocal cases (22.2%) in ^18^F-FDG PET/CT had positive BMI.

^18^F-FDG PET/CT can replace routine BMB when there is frank BMI like in (focal or heterogenous BM uptake), so there is no need for further BMB in these cases. Additionally, ^18^F-FDG PET/CT can guide the site for biopsy in cases with patchy BM infiltration to avoid false negative results. So, ^18^F-FDG PET/CT should be performed before BMB to detect the active site for biopsy.

Vishnu et al. examined 99 cases with DLBCL. Among these cases, 38% exhibited BMI. 24% of the total number of cases was found to have positive results for BMI when using ^18^F-FDG PET/CT, while only 14% had positive results when using BMB. BMB found only two cases (2%) that were not identified by ^18^F-FDG PET/CT, while ^18^F-FDG PET/CT detected 12 patients (12%) who were negative, according to BMB (Vishnu et al. 2017). This finding is comparable to our results in which ^18^F-FDG PET/CT detected 12 positive cases (18%), and 3 cases were negative by BMB as biopsy was not taken from the active site. The agreement between ^18^F-FDG PET/CT scans and also BMB yielded a concordance rate of 85 (86%) compared to 39/66 patients (59%) in our study and discordance between both modalities in 14 patients (14%) compared to 27/66 patients (41%) in our study.

In a study done by Elamir Y et al., ^18^F-FDG PET/CT had a better sensitivity of 95.6% in identifying BMI compared to 46.7% in BMB, while both modalities exhibited excellent specificity (98% vs. 100%). ^18^F-FDG PET/CT scans were more accurate than BMB (97.2% vs. 83.4%) (Elamir et al. 2020).

The findings of our study demonstrated that ^18^F-FDG PET/CT was more accurate (85% vs. 59%) and more specific (86% vs. 53%) than BMB. However, the sensitivity of both modalities was comparable (80%). The increased sensitivity of BMB was due to the technique used with bilateral iliac bone marrow biopsy instead of conventional unilateral biopsy to decrease the false negative results.

These results contradict the meta-analysis conducted by Pakos et al. (Pakos et al. 2005), which involved 587 patients. The findings from the ^18^F-FDG PET/CT studies did not demonstrate a strong agreement with those of BMB in diagnosing BMI. The ^18^F-FDG PET/CT demonstrated a sensitivity of 51% (95% CI, 38–64%) and a specificity of 91% (95% CI, 85–95%). Only the BMB was utilized as the benchmark; hence, ^18^F-FDG PET/CT was not suggested as a substitute for the regular BMB in this research.

In the current study, ^18^F-FDG PET/CT tends to be more sensitive, specific, and accurate in HL than in NHL. However, the diagnostic performance of BMB is better in NHL than in HL. These results are consistent with those reported by Kandeel et al., who revealed that ^18^F-FDG PET/CT demonstrated better diagnostic accuracy than BMB in detecting BMI in patients with HL (Kandeel et al. 2020). .

Our research yielded fair agreement (Kappa 0.33) between both modalities in patients with HL while moderate agreement in NHL patients (kappa 0.45). These results could be due to the low number of cases with NHL in our study. Our findings agree with the research conducted by Cistaro et al., who similarly observed fair agreement between both modalities in pediatric patients with HL (kappa was 0.398) with *p*-value < 0.001, (Cistaro et al. 2018).

Ann Arbor criteria classify the disease as stage IV based on hematogenous dissemination rather than direct extension, with BMI as a determining factor for this upgrade. Upstaging of 2/66cases (3%)from stage II and III to stage IV with change in management by ^18^F-FDG PET/CT through detection of BMI and in further 11/66 cases with stage IV by nodal and/or extra-nodal affection, the stage was confirmed by detection of BMI in ^18^F-FDG PET/CT. These findings were consistent with those of Berthet et al., who investigated the impact of both modalities on determining disease stage and change in management. In 11/133 patients (< 10%), ^18^F-FDG PET/CT led to upstaging to stage IV and modifications in treatment approach in 4 patients (3.0%) (Berthet et al. 2013). A study conducted by Khan et al. reported comparable findings (Khan et al. 2013).

The utilization of both modalities is complementary in evaluating BMI (Kand et al. 2010). When lesions appear outside the typical regions for iliac BMB, ^18^F-FDGPET/CT can be used to determine the biopsy location based on metabolically active sites. Several studies indicate that performing regular BMB is essential when ^18^F-FDG PET/CT scan shows a negative result. This is because 18F-FDG PET/CT scans may not always detect early or limited infiltration of the bone marrow, which can be identified through BMB (Cortés-Romera et al. 2014).In our study we concluded that ^18^F-FDG PET/CT should be done before BMB and may substitute BMB in positive cases with focal or patchy BM uptake and in negative cases. However, BMB is complementary to ^18^F-FDG PET/CT in equivocal cases with unexplained diffuse homogenous BM uptake greater than liver uptake to differentiate between BMI and reactive hyperplasia.

### Limitations

We incorporated a heterogeneous group of patients with HL and NHL. The current sample size in different subtypes of HL and NHL was insufficient to assess the diagnostic performance of ^18^F-FDG PET/CT and BMB in detecting BMI in each subtype separately. Therefore, a bigger sample size is required in future research to assess each subtype separately.

## Conclusions

^18^F-FDG PET/CT detects BMI during the initial staging of pediatric lymphoma more accurately than routine BMB. ^18^F-FDG PET/CT should be performed before BMB to detect the active site for biopsy in cases with focal/patchy BM infiltration to avoid false negative results.

In certain equivocal cases with unexplained diffuse BM uptake, BMB is required as a complementary method to differentiate between BMI and BM reactive hyperplasia.

^18^F-FDG PET/CT has high concordance to BMB, so there is no need for further BMB to exclude BMI after a negative ^18^F-FDG PET/CT study.

## Data Availability

All data used in this study can be made available on request.

## Abbreviations

PL: Pediatric lymphoma
HL: Hodgkin’s lymphoma
NHL: Non-Hodgkin’s lymphoma
BM: Bone marrow
BMB: Bone marrow biopsy
BMI: Bone marrow infiltration
PET/CT: Positron emission tomography/computed tomography
CT: Computed tomography
MRI: Magnetic resonance imaging
